# Oseltamivir Improved Thrombocytopenia During Veno-Arterial Extracorporeal Membrane Oxygenation in Adults With Refractory Cardiac Failure: A Single-Center Retrospective Real-World Study

**DOI:** 10.3389/fcvm.2021.645867

**Published:** 2021-07-26

**Authors:** Yuan Li, Lin Wang, Jianning Zhang, Hui Han, Han Liu, Chaoyang Li, Haipeng Guo, Yuguo Chen, Xiaomei Chen

**Affiliations:** ^1^Department of Critical Care Medicine, Qilu Hospital, Cheeloo College of Medicine, Shandong University, Jinan, China; ^2^Department of Hematology, Qilu Hospital, Cheeloo College of Medicine, Shandong University, Jinan, China; ^3^Department of Emergency, Qilu Hospital, Cheeloo College of Medicine, Shandong University, Jinan, China

**Keywords:** extracorporeal membrane oxygenation, veno-arterial, thrombocytopenia, platelets, cardiac failure, oseltamivir, desialylation

## Abstract

**Background:** Severe thrombocytopenia is a common complication of extracorporeal membrane oxygenation (ECMO). Oseltamivir can be used to treat infection-associated thrombocytopenia.

**Objective:** To evaluate the effect of oseltamivir on attenuating severe thrombocytopenia during ECMO.

**Methods:** This was a single-center real-world study in critically ill patients supported with venous-arterial extracorporeal membrane oxygenation (VA-ECMO). Patients suspected or confirmed with influenza received oseltamivir according to the Chinese guidelines. Thrombocytopenia and survival were compared between the oseltamivir-treated and untreated group. The factors associated with survival were analyzed by multivariable Cox analysis.

**Results:** A total of 82 patients were included. All patients developed thrombocytopenia after initiating VA-ECMO. Twenty-three patients received oseltamivir (O^+^ group), and 59 did not use oseltamivir (O^−^ group). During the first 8 days after VA-ECMO initiation, the platelet count in the O^+^ group was higher than that in the O^−^ group (all *P* < 0.05). The patients in the O^+^ group had a higher median nadir platelet count (77,000/μl, 6,000–169,000/μl) compared with the O^−^ group (49,000/μl, 2,000–168,000/μl; *P* = 0.04). A nadir platelet count of <50,000/μl was seen in 26% of the patients in the O^+^ group, compared with 53% in the O^−^ group (*P* = 0.031). No significant difference in survival from cardiac failure was seen between the O^+^ and O^−^ group (48 vs. 56%, *P* = 0.508). The Sequential Organ Failure Assessment (SOFA) score on initiation of VA-ECMO were independently associated with survival (OR = 1.12, 95% confidence interval (95% CI): 1.02–1.22, *P* = 0.015).

**Conclusions:** Oseltamivir could ameliorate VA-ECMO-related thrombocytopenia. These findings suggested the prophylactic potential of oseltamivir on severe thrombocytopenia associated with the initiation of VA-ECMO.

## Background

ECMO is increasingly used as a rescue therapy for patients with refractory cardiac/respiratory failure to provide temporary support or a bridge in the management decision-making for adult and pediatric patients (1–4). There were limited clinical trial data supporting ECMO use in adults, but it was shown that ECMO might reduce mortality compared to conventional ventilation (1–4).

Platelets are activated and even impaired due to biological incompatibility and high shear stress within 15 min after starting ECMO. The activation or damage process continues throughout ECMO until it is discontinued (5). Severe thrombocytopenia is a common complication in patients supported with ECMO. Persistent severe thrombocytopenia can independently predict 90-day mortality (6). The recovery of platelet count over time will discriminate between survivors and non-survivors. Thus, the correction of thrombocytopenia is a key issue for clinicians during ECMO. Except for emergent platelet transfusion, there is currently no effective method to prevent severe thrombocytopenia when necessary.

Nevertheless, a better understanding of platelet function and biology could yield some hints to improve platelet levels during ECMO. Indeed, recent studies have highlighted the role of platelet desialylation in platelet clearance. Platelet desialylation exposes its β-galactose residues, which can be recognized by Ashwell-Morell receptors (AMRs) on hepatocytes, leading to platelet phagocytosis in the liver (7). Furthermore, platelet desialylation can be induced by exogenous neuraminidases from pathogens (8) and by intracellular neuraminidase (Neu1) translocation to the platelet surface during platelet activation (9). Therefore, desialylation could be responsible, at least in part, for the pathogenesis of thrombocytopenia in many diseases and the clearance of transfused platelets remaining in the circulation (10). It has been shown that inhibition of platelet desialylation can extend the lifespan of circulating platelets and increase their overall number (9, 11).

Oseltamivir is an antiviral agent commonly used to prevent and treat influenza A and B. It is a viral sialidase inhibitor that prevents the release of progeny virions (12). Oseltamivir can treat infection-associated thrombocytopenia by inhibiting platelet desialylation (13, 14). Therefore, this study was designed to evaluate the effect of oseltamivir on attenuating severe thrombocytopenia during ECMO. The results could provide a novel method to prevent ECMO-related thrombocytopenia in patients who were already in critical condition.

## Methods

### Study Design and Patients

This was a single-center real-world study conducted in critically ill patients receiving VA-ECMO support to evaluate whether oseltamivir administration would ameliorate VA-ECMO-related thrombocytopenia. This study was conducted in accordance with the principles of the Declaration of Helsinki, and was approved by the Medical Ethics Committee of Qilu Hospital of Shandong University before the study began. The informed consent form was signed by the patients or their legal representatives.

Adults (≥18 years) who received VA- ECMO for more than 24 h in the intensive care unit (ICU) of Qilu Hospital of Shandong University from May 2016 to July 2020 were included. The patients were diagnosed with cardiac failure due to cardiogenic shock (CS), cardiac arrest (CA), or acute right ventricular dysfunction. Day (D) 0 was defined as the day when VA-ECMO was initiated. None of the enrolled patients showed thrombocytopenia on D0 just before cannulation. Before and during VA-ECMO support, the patients with suspected or confirmed influenza infection were given oseltamivir phosphate (Kewei, Yichang East Sunshine Changjiang Pharmaceutical, China, H20065415) orally or through a feeding tube, once every 12 h, 75 mg each time for 10 days.

According to the guidelines for the diagnosis and treatment of influenza developed by the Ministry of Health of the People's Republic of China (2011 edition), the course of oseltamivir treatment for severely ill patients could be extended to 10 days. Influenza was diagnosed with a rapid serum antigen detection method.

### Data Collection

For each enrolled patient, the following variables were collected: (1) general characteristics including age, sex, and comorbidities; (2) severity of illness assessed by Acute Physiology and Chronic Health Evaluation (APACHE) II (15) and Sequential Organ Failure Assessment (SOFA) (16) scores; (3) laboratory examination data; (4) interventions including continuous renal replacement therapy (CRRT) and oxygenator circuit changes; (5) comorbidities such as disseminated intravascular coagulation (DIC), heparin-induced thrombocytopenia (HIT), sepsis, hepatic dysfunction [defined as a ≥2-fold increase in alanine aminotransferase (17)], acute kidney injury (defined as a ≥1.5-fold increase in creatinine) (18), bleeding and thrombotic events; and (6) doses of platelet transfusions (in units of platelets).

### ECMO

Based on the patient's blood vessel diameter and estimated cardiac output, the ECMO cannula size was 21–23 Fr for venous drainage and 15–17 Fr for arterial reinfusion. No bicaval dual-lumen cannulas were adopted for ECMO. The Rotaflow centrifugal pump (Maquet, Rastatt, Germany) and Quadrox D or I oxygenator (Maquet, Rastatt, Germany) were used. Unfractionated heparin (100 units/kg) was given at the time of cannulation, followed by a continuous intravenous infusion of unfractionated heparin for a target activated partial thromboplastin time (aPTT) of 50–70 s, unless there was an indication for a higher level of anticoagulation.

### Platelet Transfusions

During ECMO, prophylactic platelet transfusion was given when the platelet count was <50,000/μl. Patients presenting with active hemorrhage needed platelet transfusion when the platelet count was lower than 100,000/μl.

### Statistical Analysis

Statistical analyses were conducted using SPSS 17.0 (IBM, Armonk, NY, USA) and GraphPad Prism 7 (GraphPad Software Inc., San Diego, CA, USA). The continuous variables were presented as medians [interquartile range (IQR)] or means ± standard deviations (SD), based on data normality distribution analyzed using the Kolmogorov-Smirnov test. The continuous variables were analyzed using the Mann-Whitney *U*-test. The categorical data are presented as *n* (%) and were analyzed using the chi-square test. Survival analysis was performed using the Kaplan-Meier method, and the curves were compared using the log-rank test. A multivariable Cox proportional hazards regression model was used to evaluate the predictors of survival from cardiac failure. All tests were two-sided. A *P*-value of < 0.05 was considered statistically significant.

## Results

### Characteristics of the Patients

**Figure 1:**
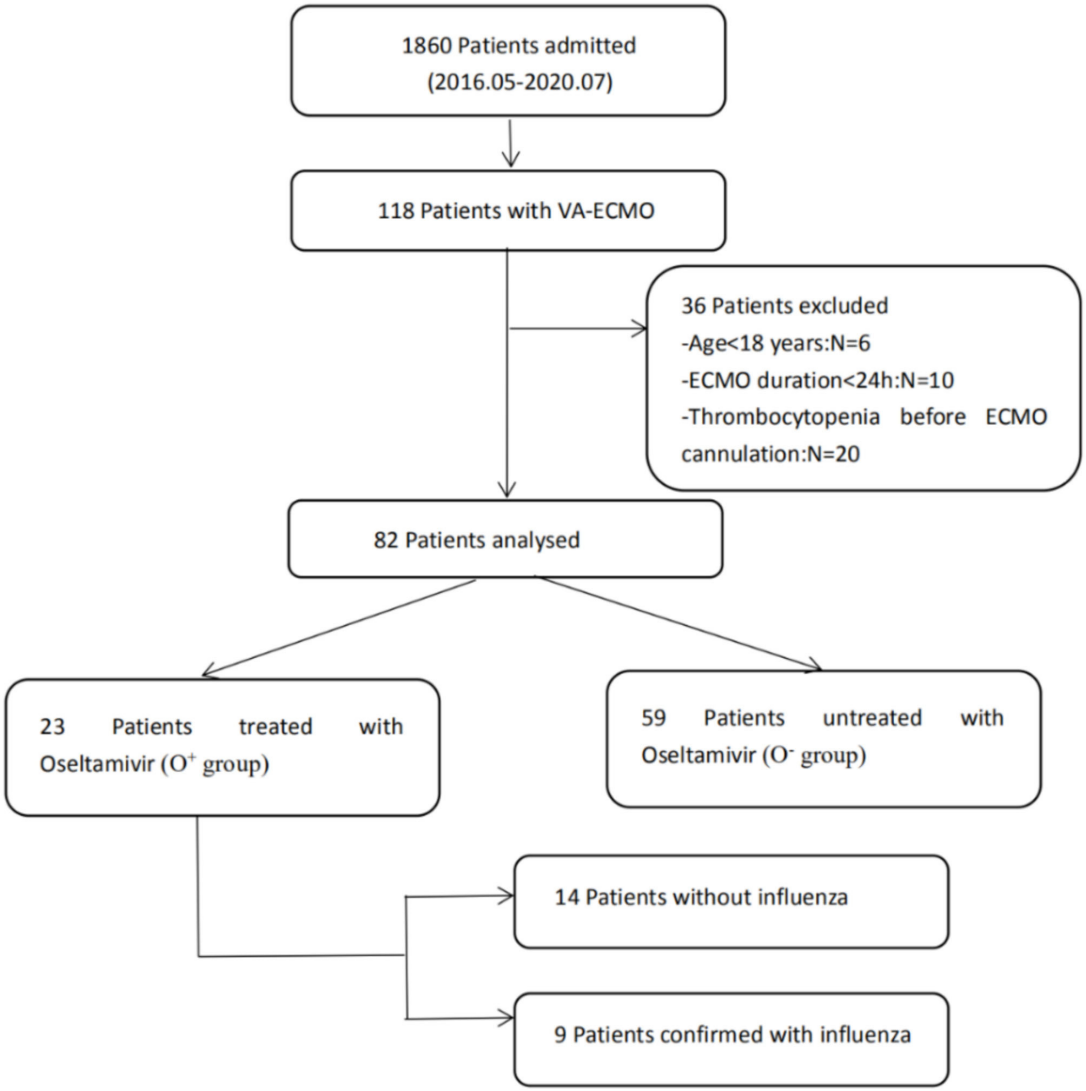
Patient recruitment flowchart. ECMO, extracorporeal membrane oxygenation.

A total of 82 patients without thrombocytopenia before cannulation were included (Figure 1). They all received VA-ECMO support due to refractory cardiac failure, including 59 patients with CS, 13 patients with CA, and 10 patients with acute right ventricular dysfunction. Among them, 23 patients were suspected of influenza infection with flu-like symptoms, including cough, fatigue, sore throat, headache, and fever. Despite the confirmation with influenza infection in only nine patients by rapid serum antigen detection, all patients received a full course of oseltamivir anti-influenza treatment empirically, from 2 to 3 days before VA-ECMO until 7–8 days after VA-ECMO, for a total of 10 days, because they were admitted during the seasonal influenza pandemic. The clinical characteristics of the oseltamivir-treated patients (O^+^ group) and untreated patients (O^−^ group) were described in Table 1. No differences in any demographic variables were found between the two groups. The APACHE II score, SOFA score, and baseline platelet count at VA-ECMO initiation were not statistically different between the two groups. One patient in the O^+^ group developed HIT on day 12 and was successfully weaned from VA-ECMO on day 14.

**Table 1:** Characteristics of adults who received VA-ECMO for refractory cardiac failure (*n* = 82).

**Variables**	**Oseltamivir- untreated group** **(***n*** = 59)**	**Oseltamivir-treated group** **(***n*** = 23**)	***P***
Age	55 (10-81)	47 (13–76)	0.060^a^
Female, *n* (%)	19 (32)	6 (26)	0.589^b^
Days on ECMO treatment	8 (6-15)	10 (7–13)	0.399^a^
APACHE II	26 ± 8	27 ± 5	0.682^a^
SOFA	11 ± 3	10 ± 4	0.072^a^
Platelet counts on day 1 before cannulation (× 10^3^/μl)	179 (134-247)	199 (144–278)	0.329^a^
Complications during the first 8 days of ECMO treatment	5 (8)	2 (3)	0.531^b^
Sepsis, *n* (%)			
Hepatic dysfunction, *n* (%)	3 (5)	4 (17)	0.073^b^
CRRT, *n* (%)	37 (62)	3 (9)	0.832^b^
DIC, *n* (%)	17 (29)	2 (9)	0.079^b^
Circuit exchange, *n* (%)	3 (5)	1 (4)	0.832^b^
Thrombosis, *n* (%)	0 (0)	0 (0)	0.324^b^
HIT, *n* (%)	0 (0)	0 (0)	0.324^b^

*Continuous data are presented as mean ± standard deviation or median (interquartile range). VA-ECMO, venous-arterial extracorporeal membrane oxygenation; APACHE II, Acute Physiology and Chronic Health Evaluation II; SOFA, Sequential Organ Failure Assessment; CRRT, continuous renal replacement therapy; DIC, disseminated intravascular coagulation; HIT, heparin-induced thrombocytopenia*.

a *Mann-Whitney U-test*.

b *Fisher exact or chi-square test*.

### Clinical Outcomes

**Figure 2:**
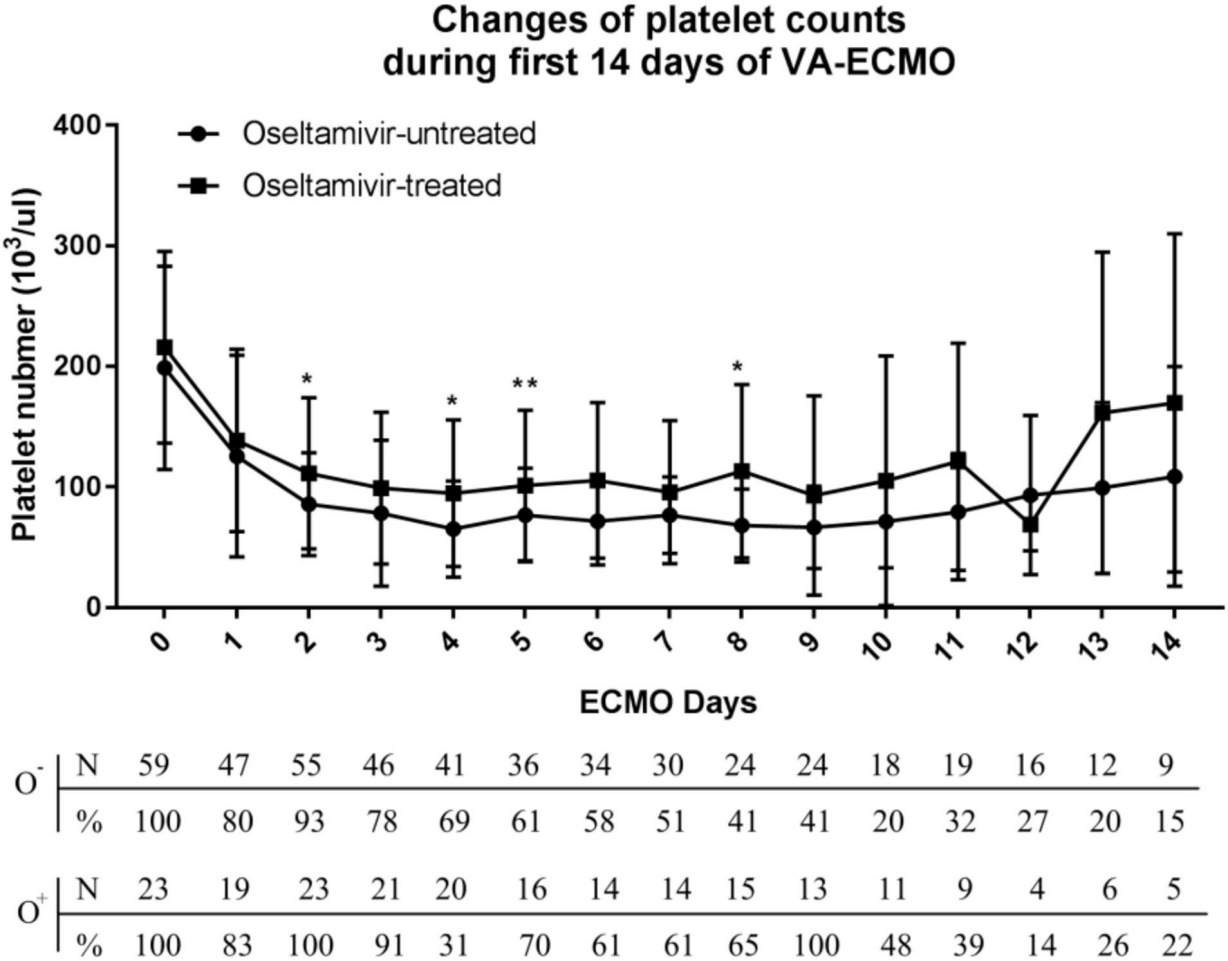
Variation of platelet counts in the oseltamivir-treated and oseltamivir–untreated groups during the first 14 days of veno-arterial extracorporeal membrane oxygenation (VA-ECMO) support.

Overall, 85% of the patients had thrombocytopenia and 44% developed severe thrombocytopenia (platelet count <50,000/μl). As shown in Figure 2, a decrease in platelet count was observed within <24 h after initiating VA-ECMO. The nadir platelet count was observed on day 4, and the platelet started to increase on day 8. The biphasic temporal pattern was seen in both groups (Figure 2).

During the first 8 days after VA-ECMO initiation, the platelet count in the O^+^ group was higher than that in the O^−^ group on day 2 (113,000 ± 62,700 vs. 85,700 ± 42,800/μl, *P* = 0.04), day 4 (94,800 ± 60,800 vs. 65,000 ± 39,800/μl, *P* = 0.02), day 6 (105,500 ± 64,600 vs. 71,500 ± 36,100/μl, *P* < 0.001), and day 8 (113,000 ± 71,800 vs. 68,000 ± 30,400/μl, *P* = 0.01). The patients in the O^+^ group had a higher median nadir platelet count (77,000/μl, 6,000–169,000/μl), compared with the O^−^ group (49,000/μl, 2,000–168,000/μl; *P* = 0.04). A nadir platelet count <50,000/μl was seen in 26% of the patients in the O^+^ group, compared with 53% in the O^−^ group (*P* = 0.031). There was no difference in platelet count recovery time between the two groups. Platelet transfusions were administered in 4% of the patients in the O^+^ group, compared with 32% in the O^−^ group (*P* = 0.022), and the O^+^ group received a smaller amount of platelets (0.09 ± 0.42 vs. 0.95 ± 2.41 apheresis units; *P* = 0.007). There was a tendency toward fewer bleeding events in the O^+^ group compared with the O^−^ group (9 vs. 29%, *P* = 0.052) (Table 2). In the O^+^ group, there was no difference in platelet count changes regardless of influenza infection status (*P* > 0.05) (Table 3).

**Table 2:** Changes in platelet count and treatment outcomes in patients with refractory cardiac failure during the first 8 days of ECMO.

**Variables**	**Oseltamivir-untreated group** **(***n*** = 59**)	**Oseltamivir-treated group** **(***n*** = 23)**	***P***
Nadir Platelet count (× 10^3^/μl)	49 (31–66)	77 (48–102)	0.040^a^
Day to nadir platelet	4 (3–6)	4 (2–7)	0.657^a^
Percentage of patients with nadir platelet <50 × 10^3^/μl, *n* (%)	31 (53%)	6 (26)	0.031^b^
Percentage of patients with nadir platelet <100 × 10^3^/μl, *n* (%)	50 (85)	17 (74)	0.254^b^
Recovery time to platelet ≥100 × 10^3^/μl (days)	4 (2–6)	3 (0–5)	0.134^a^
Administration of platelet transfusion (apheresis unit), *n* (%)	19 (32%)	1 (4%)	0.022^b^
Platelet transfusion times	1 (0–15)	0 (0–2)	0.002^a^
Amount of platelet transfusion	1 (0–17)	0 (0–2)	0.007^a^
Bleeding, *n* (%)	17 (29)	2 (9)	0.052^b^
Survival fromcardiac failure, *n* (%)	33 (56)	11 (48)	0.508^b^

*Continuous data are presented as median (interquartile range). ECMO, extracorporeal membrane oxygenation*.

a *Mann-Whitney U-test*.

b *Fisher exact or chi-square test*.

**Table 3:** Characteristics of the patients treated with oseltamivir during hospital stay (*n* = 23).

**Variables**	**Influenza** **(***n*** = 9)**	**No influenza** **(***n*** = 14)**	***P***
Days on ECMO treatment	13 (11–15)	9 (5–10)	0.014^a^
Hospital stay (days)	21 (15–29)	11 (5–26)	0.185^a^
APACHE II	27 ± 4	28 ± 6	0.165^a^
SOFA	9 ± 3	11 ± 5	0.776^a^
Platelet counts on d1 before cannulation (× 10^3^/μl)	147 (114–321)	221 (173–254)	0.777^a^
Nadir platelet count (× 10^3^/μl)	77 (50–97)	78 (35–101)	0.753^a^
Day to nadir PLT	6 (3–7)	4 (2–5)	0.237^a^
Percentage of patients with nadir platelet <50 × 10^3^/μl, *n* (%)	5 (56%)	3 (23%)	0.708^b^
Percentage of patients with nadir platelet <100 × 10^3^/μl, *n* (%)	6 (67%)	9 (69%)	0.537^b^
Recovery time to platelet ≥100 × 10^3^/μl (days)	4 (1–6)	3 (0–5)	0.481^a^
Administration of platelet transfusion (apheresis units), *n* (%)	1 (11)	0 (0)	0.391^b^
Platelet transfusion times, *n* (%)	0 (0–2)	0 (0–0)	0.212^a^
Amount of platelet transfusion	0 (0–2)	0 (0–0)	0.212^a^
Survival from cardiac failure, *n* (%)	5 (54)	6 (43)	0.433^b^

*Continuous data are presented as mean ± standard deviation or median (interquartile range). ECMO, extracorporeal membrane oxygenation; APACHE II, Acute Physiology and Chronic Health Evaluation II; SOFA, Sequential Organ Failure Assessment; CRRT, continuous renal replacement therapy; DIC, disseminated intravascular coagulation; HIT, heparin-induced thrombocytopenia*.

a *Mann-Whitney U-test*.

b *Fisher exact or chi-square test*.

### Prognosis Analysis

**Figure 3:**
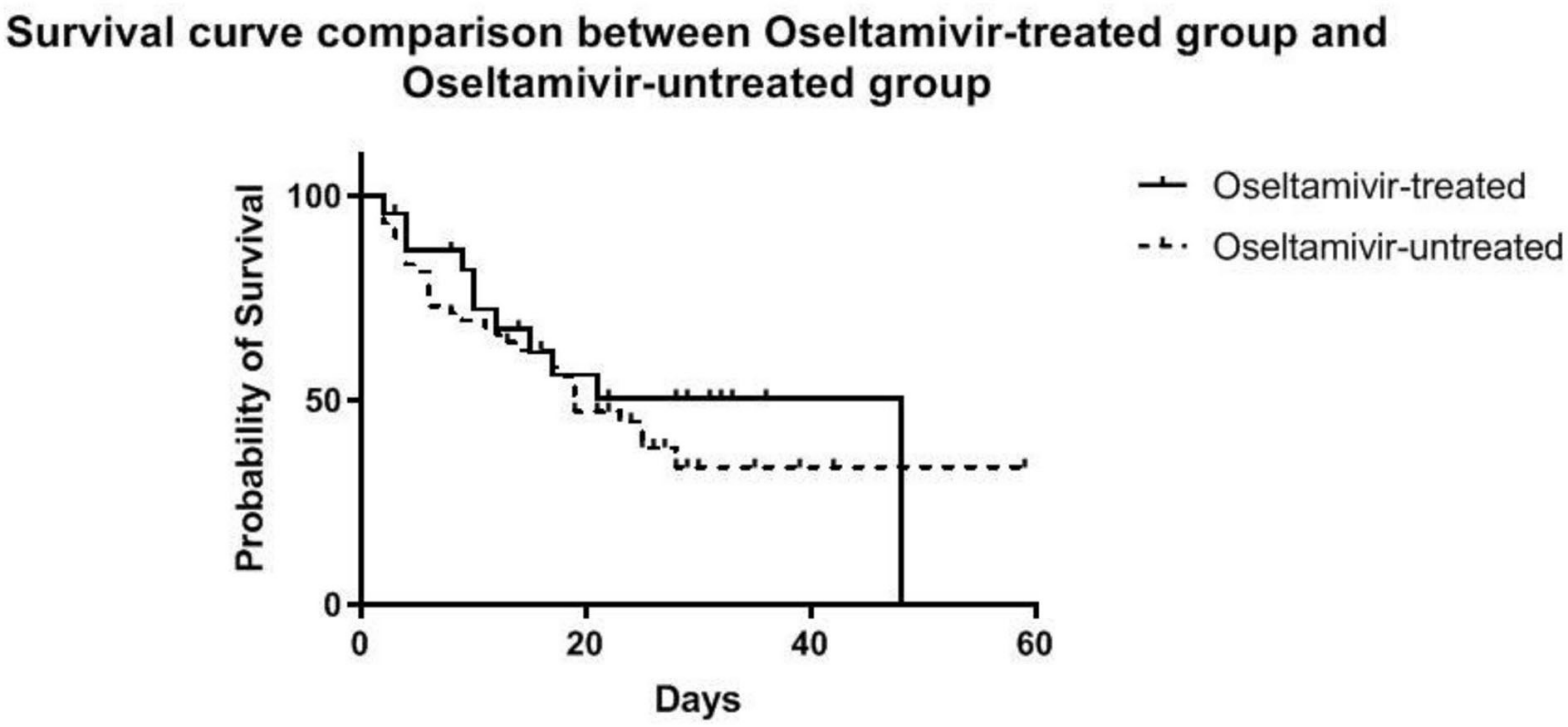
Estimated survival of patients during VA-ECMO support until the time of discharge, stratified into the oseltamivir -treated and -untreated group.

No significant difference in survival from cardiac failure was seen between the O^+^ and O^−^ group (48 vs. 56%, *P* = 0.508) (Figure 3). As shown in Table 4, among the variables possibly associated with survival, only the SOFA scores at VA-ECMO initiation were independently associated with survival (OR = 1.12, 95% CI: 1.02–1.22, *P* = 0.015).

**Table 4:** Multivariate Cox proportional hazard analysis of factors influencing survival in all patients who received ECMO.

**Variables**	**Hazard ratio**	**95% CI**		***P***
		**Lower**	**Upper**	
Univariable analysis				
Age	1.017	0.998	1.037	0.083
Female	0.783	0.395	1.555	0.485
Oseltamivir treat	0.796	0.401	1.579	0.513
APACHE score	1.035	0.991	1.081	0.123
SOFA score	1.11	1.022	1.204	0.013
Sepsis	1.052	0.407	2.717	0.917
CRRT	0.915	0.489	1.713	0.782
DIC	1.318	0.675	2.575	0.418
Hepatic dysfunction	1.023	0.316	3.317	0.97
Multivariable analysis				
Age	1.012	0.99	1.035	0.281
Female	0.612	0.293	1.279	0.192
Oseltamivir treat	1.119	0.516	2.429	0.776
APACHE score	1.027	0.976	1.08	0.301
SOFA score	1.116	1.017	1.225	0.021
Sepsis	1.127	0.397	3.196	0.822
CRRT	0.767	0.377	1.563	0.466
DIC	1.294	0.629	2.644	0.484
Hepatic dysfunction	0.918	0.277	3.046	0.889
Final multivariable prediction model				
Age	1.014	0.993	1.035	0.199
Oseltamivir	1.051	0.509	2.168	0.893
SOFA score	1.118	1.022	1.223	0.015
CRRT	0.748	0.373	1.502	0.415

*APACHE score, SOFA score, and DIC were obtained at ICU admission. 95% CI, 95% confidence interval; ECMO, extracorporeal membrane oxygenation; APACHE II, Acute Physiology and Chronic Health Evaluation II; SOFA, Sequential Organ Failure Assessment; CRRT, continuous renal replacement therapy; DIC, disseminated intravascular coagulation; HIT, heparin-induced thrombocytopenia*.

## Discussion

Thrombocytopenia is a common complication of ECMO (6). Oseltamivir can be used to treat infection-associated thrombocytopenia (13, 14). This study aimed to evaluate the effect of oseltamivir on attenuating severe thrombocytopenia during ECMO. The results suggested that oseltamivir could ameliorate VA-ECMO-related thrombocytopenia. These findings indicated the potential prophylactical effect of oseltamivir on severe thrombocytopenia associated with the initiation of VA-ECMO. This was the first study to provide evidence that oseltamivir could prevent severe thrombocytopenia induced by VA-ECMO.

The prevailing view has been that platelets are activated by the artificial surface of ECMO and impaired by high shear stress in the extracorporeal circuit, leading to an early decline in platelet count (19–21). It could be hypothesized that platelet desialylation participated in this process through intracellular Neu1 translocation to the platelet surface, which contributed to increased clearance of the platelets. As observed in this study, the platelet count dropped dramatically after the start of VA-ECMO, and the declining trend continued until days 8–10, except for one patient in the O^+^ group who developed HIT on day 12.

Oseltamivir, as a viral neuraminidase inhibitor, has been shown to significantly increase platelet counts in a mouse model of anti-GPIba-mediated ITP by inhibiting platelet desialylation (22). Oseltamivir was also successfully used to treat an adult ITP patient who responded poorly to conventional therapies, including corticosteroids, intravenous immunoglobulin (IVIG), recombinant human thrombopoietin, rituximab, danazol, and vindesine (23). A multi-center and randomized controlled trial showed that platelet desialylation levels were increased significantly in septic patients, and the administration of oseltamivir attenuated thrombocytopenia associated with sepsis (14). Recently, a retrospective analysis showed that oseltamivir use increased the platelet counts regardless of influenza status (13), supporting the findings in the present study.

Therefore, we speculated that oseltamivir might improve ECMO-induced thrombocytopenia by inhibiting platelet desialylation. Meanwhile, platelet activation might be attenuated through suppression of desialylation, as desialylation and platelet activation were previously shown to exist in a positive feedback loop (22). In addition to the platelet-reducing effect exerted by extracorporeal circuit, critical illness severity and comorbid conditions are associated with the development of thrombocytopenia in critically ill patients (24). In this study, none of the examined complications (hepatic dysfunction, renal dysfunction, sepsis, HIT, thrombosis, and DIC) were associated with the patient's prognosis, only the SOFA score was associated with survival.

Thrombocytopenia increases the bleeding risk during ECMO, especially because of the need for anticoagulants to avoid circuit clogging, which leads to a higher threshold for platelet transfusion in patients receiving ECMO compared with other critically ill patients (25). The Extracorporeal Life Support Organization (ELSO) has recommended that the platelet count should be maintained above 100,000/μL (26). So far, platelet transfusion is the only readily available treatment for thrombocytopenia. However, platelet transfusion is extremely costly with limited resources. Moreover, the benefits of multiple platelet transfusions for patients receiving ECMO remains unknown. Platelet transfusion was shown to be associated with increased risks of thrombosis and in-hospital mortality in large retrospective studies involving patients with platelet consumptive disorders (27). Recent studies have suggested that a more restrictive platelet transfusion practice might be applicable to critically ill neonates (28, 29). In this study, oseltamivir use attenuated thrombocytopenia during VA-ECMO and reduced the requirement for platelet transfusion. A trend of reduced bleeding events was also seen in the treatment O^+^ group.

In this study, oseltamivir treatment did not affect the survival from cardiac failure, although, by day 8 after starting ECMO, significantly higher platelet counts that was considered as a predictor of good prognosis for ECMO patients after cardiac surgery (6). The reason for this contradiction might be that only a few patients received oseltamivir treatment.

This study had some limitations and the results must be interpreted with caution. First, the retrospective and observational nature that relied on the availability of recorded data made the studies prone to selection bias. Second, the relatively small number of ECMO patients treated with oseltamivir limited the statistical power of the analysis. Since oseltamivir has been shown to attenuate severe thrombocytopenia regardless of influenza status, it might be widely used in ECMO patients in future prospective clinical trials. We only evaluated the effect of oseltamivir on the prevention of severe thrombocytopenia in patients receiving VA-ECMO, and the findings might not be generalized to VV- ECMO. Third, we should be aware that there were both known and unknown confounders affecting the platelet counts. Influenza itself could cause thrombocytopenia through desialylation. Still, we could not differentiate whether oseltamivir acted on platelet neuraminidase or viral neuraminidase. Infections can induce inflammatory responses including elevated IL6, which has been shown to up-regulate hepatic thrombopoietin and increase platelet production (30). Further, research on the mechanism of oseltamivir on reducing thrombocytopenia induced by ECMO was needed.

Last, flow cytometry detection of the expression of platelet activation markers, which was considered as an alternative indicator of platelet function (31–34), was not performed in this study since platelet aggregation and adherence results needed to be interpreted with caution during thrombocytopenia (35).

## Conclusions

Oseltamivir increased the median nadir platelet count, decreased the prevalence of severe thrombocytopenia, and reduced platelet transfusions. Those results supported the clinical effect of oseltamivir in preventing VA-ECMO-induced severe thrombocytopenia. Future studies were warranted to confirm this benefit.

## Data Availability Statement

The original contributions presented in the study are included in the article/supplementary material, further inquiries can be directed to the corresponding author/s.

## Ethics Statement

The studies involving human participants were reviewed and approved by the Ethics Committee of Qilu Hospital of Shandong University. The patients/participants provided their written informed consent to participate in this study.

## Author Contributions

YL: designed the study and drafted the manuscript. LW: participated in the design of the study helped to draft the manuscript. JZ: collected the medical data. HH: performed the statistical analysis. HL: participated in coordination and helped to collect the medical data. CL: participated in the statistical analysis and data curation. HG: embellished the manuscript. YC: conceived of the study and helped to draft the manuscript. XC: proofread the manuscript. All authors read and approved the final manuscript.

## Conflict of Interest

The authors declare that the research was conducted in the absence of any commercial or financial relationships that could be construed as a potential conflict of interest.

## Publisher's Note

All claims expressed in this article are solely those of the authors and do not necessarily represent those of their affiliated organizations, or those of the publisher, the editors and the reviewers. Any product that may be evaluated in this article, or claim that may be made by its manufacturer, is not guaranteed or endorsed by the publisher.

## Abbreviations

ECMO: extracorporeal membrane oxygenation
VA-ECMO: venous-arterial extracorporeal membrane oxygenation
SOFA: Sequential Organ Failure Assessment
VV: veno-venous
VA: veno-arterial
AMRs: Ashwell-Morell receptors
Neu1: neuraminidase
CS: cardiogenic shock
CA: cardiac arrest
ICU: intensive care unit
D: day
APACHE: Acute Physiology and Chronic Health Evaluation
CRRT: continuous renal replacement therapy
DIC: disseminated intravascular coagulation
HIT: heparin-induced thrombocytopenia
aPTT: activated partial thromboplastin time
IQR: interquartile range
VV- ECMO: veno-venous ECMO
APACHE II: Acute Physiology and Chronic Health Evaluation II
95% CI: 95% confidence interval.
