# Corrigendum: Oseltamivir Improved Thrombocytopenia During Veno-Arterial Extracorporeal Membrane Oxygenation in Adults With Refractory Cardiac Failure: A Single-Center Retrospective Real-World Study

**DOI:** 10.3389/fcvm.2021.784904

**Published:** 2021-10-29

**Authors:** Yuan Li, Lin Wang, Jianning Zhang, Hui Han, Han Liu, Chaoyang Li, Haipeng Guo, Yuguo Chen, Xiaomei Chen

**Affiliations:** ^1^Department of Critical Care Medicine, Qilu Hospital, Cheeloo College of Medicine, Shandong University, Jinan, China; ^2^Department of Hematology, Qilu Hospital, Cheeloo College of Medicine, Shandong University, Jinan, China; ^3^Department of Emergency, Qilu Hospital, Cheeloo College of Medicine, Shandong University, Jinan, China

**Keywords:** extracorporeal membrane oxygenation, veno-arterial, thrombocytopenia, platelets, cardiac failure, oseltamivir, desialylation

In the published article, there was an error in the affiliations. Instead of Qilu Hospital, Shandong University, Jinan, China, it should be:

^1^ Department of Critical Care Medicine, Qilu Hospital, Cheeloo College of Medicine, Shandong University, Jinan, China

^2^ Department of Hematology, Qilu Hospital, Cheeloo College of Medicine, Shandong University, Jinan, China

^3^ Department of Emergency, Qilu Hospital, Cheeloo College of Medicine, Shandong University, Jinan, China.

YL is affiliated with 1, LW is affiliated with 2, JZ is affiliated with 1, HH is affiliated with 1, HL is affiliated with 1, CL is affiliated with 2, HG is affiliated with 1, YC is affiliated with 3, XC is affiliated with 1.

Furthermore, Lin Wang was not included as a co-first author in the published article. This has been corrected, and a new statement has been added to indicate that Yuan Li and Lin Wang contributed equally: “These authors contributed equally to this work.”

The authors apologize for these errors and state that this does not change the scientific conclusions of the article in any way. The original article has been updated.

## Publisher's Note

All claims expressed in this article are solely those of the authors and do not necessarily represent those of their affiliated organizations, or those of the publisher, the editors and the reviewers. Any product that may be evaluated in this article, or claim that may be made by its manufacturer, is not guaranteed or endorsed by the publisher.

